# Supplementary material for: Complexome profiling of the Chlamydomonas psb28 mutant reveals TEF5 as an early PSII assembly factor
Source: Plant Cell. 2025 Mar 17;37(6):koaf055. doi: 10.1093/plcell/koaf055 (PMC12164590; doi:10.1093/plcell/koaf055)
Supplement: koaf055_Supplementary_Data [file koaf055_supplementary_data.zip › psb28_interactive.zip!/plots/intensity/qualityPlots/peptides/MRP2_Cre17.g734612.t1.1.html]

### Cre17.g734612.t1.1

Trivial name: MRP2  
SeqCoverage [median]: 8.30  
Mass: 157.30 kDa  
  
 

| Sequence | MissedCleavage | Mass [Da] | wtIntensity | muIntensity | Score |
| --- | --- | --- | --- | --- | --- |
| AEVPRR | 1 | 726.41 | 0.00 | 896.39 | 40.69 |
| AFDAENYFIQENLRR | 1 | 1884.91 | 0.00 | 854.38 | 28.98 |
| ENIRNDTFIER | 1 | 1405.69 | 0.00 | 445.90 | 62.41 |
| GSSEAVASLQR | 0 | 1103.56 | 390.29 | 663.48 | 55.55 |
| ILAMAAGQLK | 0 | 1014.59 | 0.00 | 321.67 | 68.42 |
| IVIDGINMANIK | 0 | 1299.72 | 297.15 | 1121.25 | 81.59 |
| LSIPSFEVK | 0 | 1018.57 | 0.00 | 1593.01 | 51.85 |
| LVEDTGPHASK | 0 | 1152.58 | 1931.27 | 0.00 | 67.46 |
| SGEVVAVVGR | 0 | 971.54 | 2028.06 | 1266.59 | 146.81 |
| SLVQYVTPVEK | 0 | 1261.69 | 0.00 | 656.21 | 63.28 |
| YKLETVEVSDAR | 1 | 1408.72 | 0.00 | 1348.43 | 43.99 |
|  |  |  | 4646.8 | 9167.3 |  |
